# Characterization and Genome Sequence of Arthrobacter Phage Iter

**DOI:** 10.1128/mra.00538-22

**Published:** 2022-09-06

**Authors:** Kathleen A. Daly, Audrey E. Nesbit, Jacob D. Buchholz, Alison E. Kanak

**Affiliations:** a Department of Biology, University of North Georgia, Dahlonega, Georgia, USA; Loyola University Chicago

## Abstract

Arthrobacter phage Iter was isolated in North Georgia. Its genome is 43,963 bp with 70 open reading frames (ORFs) and a GC content of 67.4%. It shares 89.11% nucleotide identity with *Arthrobacter* phage Phives. Actinobacteriophages that share over 50% nucleotide identity are sorted into clusters, with Iter in cluster AZ.

## ANNOUNCEMENT

There is growing concern about untreatable infections resulting from antibiotic-resistant bacteria that could be addressed through the use of phage therapy. The discovery and characterization of these viruses are important basic science that can be used to address clinical needs. Here, we present the *Arthrobacter* phage Iter, isolated using a Science Education Alliance-Phage Hunters Advancing Genomics and Evolutionary Science (SEA-PHAGES) protocol (1).

**FIG 1:**
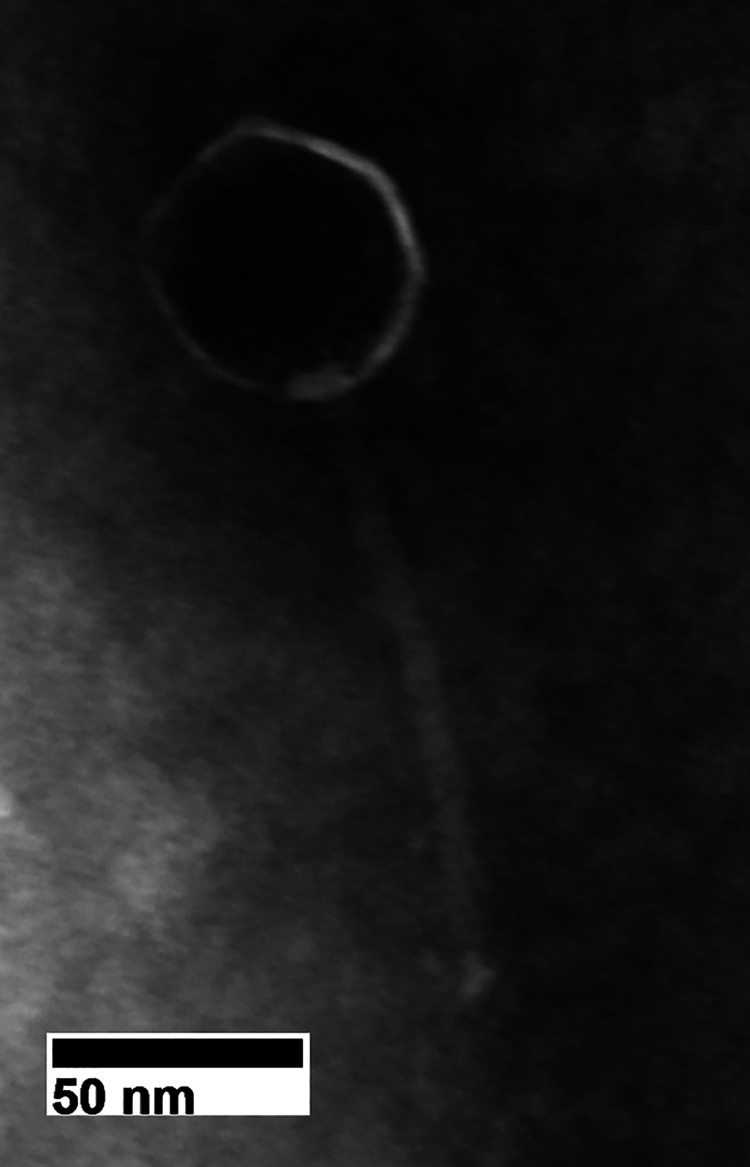
Transmission electron micrograph of Iter. TEM images were obtained using a JEM-1011 TEM (JEOL, Inc., Tokyo, Japan) at the University of Georgia Electron Microscopy facility.

Iter was isolated from soil collected in Georgia (34.470714°N, 83.975651°W) in 2022. Arthrobacter globiformis B-2979 was the host used for Iter isolation. Briefly, 7H9 liquid medium was added to soil and incubated at 37°C for 24 h. The sample was filtered using a 0.22-μm filter. Phage presence was confirmed and purified via plaque assay and amplified to a high titer to extract phage genomic DNA. Electron microscopy of Iter identified it as a *Siphoviridae* with a capsid measuring 49.03 nm in diameter and tail measuring 123.74 nm long (Fig. 1). Iter formed plaques 4 mm in diameter with distinct margins featuring a “bull's-eye” characteristic. Actinobacteriophages that share over 50% nucleotide identity are sorted into clusters and then further into subclusters where appropriate (2, 3). Iter was classified into cluster AZ.

Phage genomic DNA was extracted from Iter lysates with a Wizard DNA extraction kit (Promega) per the manufacturer’s instructions. A NEBNext Ultra II FS kit with dual-indexed barcoding was used to assemble a sequencing library. The library was run on an Illumina MiSeq system. There were 219,616 single-end 150-bp reads from the Iter library that provided 713-fold coverage of the Iter genome. These raw reads were assembled using Newbler v2.9 with default settings. The resulting single-phage contig was checked for completeness, accuracy, and phage genomic termini by using Consed v29 (4). The genome is 43,963 bp with a GC content of 67.4%.

The genome was annotated using GeneMark v3.25 (5), NCBI BLAST v2.9.0 (6), Glimmer v3.02 (7), HHpred v3.2.0 (8), ARAGORN v1.2.38 (9), and Phamerator (https://phamerator.org). Default parameters were used for all software unless otherwise specified. Hits with E values of 10e-10 or less were considered acceptable. Phamerator and GeneMark indicate that Iter has 70 open reading frames (ORFs), and 36 have predicted functions. Genes 1 to 37, 39 to 49, and 51 to 69 are transcribed in the forward direction, while genes 38, 50, and 70 are transcribed in the reverse direction. Iter is predicted to be a temperate phage, as a serine integrase (ORF51) was identified. A phamily was determined using Phamerator by using “pairwise comparisons to generate gene relationships” (10). As observed in other phamily members, Iter is predicted to code for a Cas4 family exonuclease (ORF26), which may play a role in nucleotide metabolism or CRISPR activity (11). Iter has 3′ sticky ends, with an 11-bp overhang. ORF14 and ORF15 encode tail chaperone proteins, which overlap a −1 frameshift predicted in ORF15 at nucleotide 10407, frequently observed in bacteriophages across many clusters. Iter is genetically most similar to Phives (GenBank accession no. MT889376), with 89.11% nucleotide identity via BLAST alignment.

### Data availability.

Information on Iter’s genome can be found in GenBank under accession no. ON208833. Sequencing reads are part of the Sequence Read Archive with accession no. SRX15121735 under BioProject accession no. PRJNA488469.
